# Tumor-infiltrating B cells inhibit nasopharyngeal carcinoma metastasis

**DOI:** 10.1038/s41419-026-08940-6

**Published:** 2026-06-02

**Authors:** Jing Cai, Xiangzhou Tan, Xinglong Liu, Juan Feng, Xiangying Deng, Lin Zhao

**Affiliations:** 1 Department of Pathology, The Second Xiangya Hospital, Central South University, Changsha, China; 2 Hunan Clinical Medical Research Center for Cancer Pathogenic Genes Testing and Diagnosis, Changsha, China; 3 Department of General Surgery, National Clinical Research Center for Geriatric Diseases (Xiangya Hospital), Central South University, Changsha, China; 4 Institute of Medical Sciences, National Clinical Research Center for Geriatric Diseases (Xiangya Hospital), Central South University, Changsha, China

**Keywords:** Tumour immunology, Experimental models of disease

## Abstract

Nasopharyngeal carcinoma (NPC) in advanced stages often has a poor prognosis due to distant metastasis, yet the distribution characteristics and anti-metastatic mechanisms of tumor-infiltrating B cells (TIL-B) remain unclear. In this study, single-cell RNA sequencing combined with multiplex immunofluorescence revealed that B cells were highly enriched in non-metastatic NPC and formed complete tertiary lymphoid structures (TLS), whereas TLS was absent in metastatic NPC. Functional assays in vitro and in vivo showed that TIL-B and their conditioned medium significantly inhibited NPC cell proliferation, migration, and epithelial-mesenchymal transition (EMT), while inducing apoptosis; in humanized mouse models, TIL-B suppressed subcutaneous tumor growth and lung metastasis, whereas B cell depletion accelerated tumor progression. Mechanistically, TIL-B secreted IL-12, IFN-γ, TNF-α, and CXCL13. Among them, IL-12 upregulated E-cadherin and downregulated N-cadherin and Vimentin, thereby blocking EMT and inhibiting vasculogenic mimicry (VM); CXCL13 may promote TLS formation and enhance local immune responses. Through the dual pathways of “ IL-12-EMT/VM inhibition” and “CXCL13-TLS construction,” TIL-B cooperatively restrains tumor invasion and metastasis, building a multidimensional immune defense system. This study demonstrates that TIL-B play an important regulatory role in anti-tumor immunity in NPC and suggests their therapeutic potential, providing a rationale for the development of B cell-based or TLS-targeted immunotherapeutic strategies.

## Introduction

NPC is a malignant tumor originating from the epithelial lining of the nasopharynx, characterized by marked geographic and ethnic distribution differences, with a high incidence in southern China and Southeast Asia [1]. Its occurrence and progression are closely associated with persistent Epstein-Barr virus (EBV) infection, genetic susceptibility, and environmental factors [1]. Although radiotherapy combined with chemotherapy has significantly improved survival rates in early- and mid-stage patients, advanced and metastatic NPC remains prone to recurrence, and distant metastasis is the leading cause of mortality [1]. Recent studies have shown that NPC is driven not only by genetic and signaling abnormalities in tumor cells themselves, but also by the complex immune regulation of the tumor microenvironment (TME) [2, 3].

The TME consists of tumor cells, stromal cells, vascular endothelial cells, immune cells, and extracellular matrix components. In the immune microenvironment of NPC, the roles of T cells, NK cells, dendritic cells, and macrophages have been extensively reported, while B cells—an essential component of adaptive immunity—have gained increasing attention in recent years [4, 5]. TIL-B can mediate humoral immunity by secreting immunoglobulins and can also influence antitumor responses through multiple pathways, including antigen presentation, cytokine secretion, and T cell regulation [4, 6]. However, the functions of B cells in tumors remain controversial: some studies suggest that TIL-B exert antitumor effects by promoting the formation of tumor-associated TLS, enhancing CD8⁺ T cell cytotoxicity, and secreting proinflammatory cytokines such as IL-12, IFN-γ, and TNF-α; other research indicates that regulatory B cells (Bregs) may secrete inhibitory factors such as IL-10 and TGF-β, thereby promoting immune tolerance and tumor progression[5, 7].

In NPC, studies on TIL-B remain limited. Some pathological evidence indicates that the level of B cell infiltration is associated with patient survival outcomes [8, 9], but differences in their distribution between metastatic and non-metastatic lesions, their functional status, and the mechanisms of action of their secreted factors are still unclear. Notably, B cells may indirectly interfere with tumor metastasis by inhibiting EMT [10–12]. EMT is a key process by which tumor cells acquire migratory and invasive capabilities, characterized by the downregulation of E-cadherin and the upregulation of N-cadherin and Vimentin, leading to reduced cell–cell adhesion and loss of polarity.

In recent years, TIL-B and their roles in the TME have attracted increasing attention. However, their distribution characteristics, functional mechanisms, and clinical significance in NPC remain unclear. In this study, through a combination of clinical sample analysis and in vitro and in vivo functional experiments, we revealed the potential protective role of TIL-B in NPC progression and its antitumor mechanisms mediated by cytokines, providing new theoretical evidence for immunotherapeutic strategies targeting NPC.

## Materials and methods

### Clinical sample collection

A total of six NPC patients admitted to the Second Xiangya Hospital of Central South University were enrolled in this study, including three cases in the non-metastatic group and three cases in the metastatic group. The inclusion criteria were: (1) pathologically confirmed NPC; (2) no prior systemic treatment such as radiotherapy or chemotherapy; (3) complete clinical data; and (4) signed written informed consent from the patient. The exclusion criteria were: (1) presence of other malignant tumors; (2) severe immune system diseases; and (3) insufficient specimen volume for subsequent experiments. Fresh tumor tissues were immediately placed in pre-cooled RPMI-1640 medium (containing 1% antibiotics) after surgical resection and processed within 4 h. Portions of the tissues were fixed in 10% neutral buffered formalin for 24 h, routinely dehydrated, paraffin-embedded, and used for immunohistochemical analysis of B-cell infiltration levels and the number of tumor-associated TLS. The clinical information of the patients is shown in Table S1.

### Single-cell RNA sequencing data acquisition and analysis

The single-cell RNA sequencing dataset HRA000087 was obtained from the National Genomics Data Center and includes single-cell transcriptomic data from metastatic and non-metastatic lesions of NPC patients. Data analysis was performed using the Seurat (v4.0.3) platform, following a workflow that included quality control, data normalization, batch effect correction, and t-SNE dimensionality reduction and clustering. Cell types were identified based on known marker genes. Differences in the proportion of B cells between metastatic and non-metastatic groups were statistically analyzed using the Wilcoxon rank-sum test.

### Cell culture

NPC cell lines C666-1 and HK1-EBV were preserved and provided by our laboratory. Cells were cultured in RPMI-1640 medium (Gibco, 11875-093) supplemented with 10% fetal bovine serum (FBS, Gibco, 10099-141) and 1% penicillin–streptomycin mixture (Gibco, 15140-122), and maintained at 37 °C in a humidified atmosphere containing 5% CO₂. The culture medium was replaced every other day, and when the cell density reached 80–90% confluence, cells were passaged using 0.25% trypsin-EDTA solution for digestion.

### Isolation and identification of TIL-B

Fresh NPC tissues were cut into small pieces and digested in a solution containing type I collagenase (1 mg/mL, C0130, Sigma) and DNase I (0.1 mg/mL, DN25, Sigma) at 37 °C with shaking for 30 min. The digested suspension was passed through a 70 μm cell strainer, and mononuclear cells were isolated by Ficoll-Paque Plus density gradient centrifugation (400 × *g*, 20 min, room temperature). CD20⁺ B cells were then purified using human CD20 MicroBeads (Miltenyi Biotec, 130-091-104) via magnetic-activated cell sorting (MACS). The purity was assessed by flow cytometry based on the proportion of CD19/CD20 double-positive cells, ensuring a purity greater than 95%.

### Preparation of TIL-B conditioned medium

The purified TIL-B cells were seeded at a density of 1 × 10⁶ cells/mL in complete RPMI-1640 medium supplemented with 10% FBS and incubated at 37 °C with 5% CO₂ for 48 h. After incubation, the culture supernatant was collected and centrifuged at 1000 × *g* for 10 min to remove cells and debris, followed by sterilization through a 0.22 μm filter (Millipore, SLGP033RS). The filtered supernatant was aliquoted into sterile cryovials and stored at –80 °C, avoiding repeated freeze–thaw cycles.

### Co-culture experiment

In the direct co-culture experiment, NPC cells (5 × 10³ cells/well) were seeded into 96-well plates and cultured overnight, followed by the addition of TIL-B cells at NPC: TIL-B ratios of 1:2 or 1:1. The cells were co-cultured for 48 h, after which NPC cell proliferation was assessed. In the conditioned medium treatment experiment, NPC cells were seeded into 96-well plates, and after attachment, they were treated with 50%, 75%, or 100% TIL-B conditioned medium (diluted with fresh complete medium) for 48 h. Cell viability was then measured using the Cell Counting Kit-8.

### Migration assay

The migration assay was performed using Transwell chambers with an 8 μm pore size (Corning, 3422). A total of 5 × 10⁴ NPC cells suspended in serum-free medium were seeded into the upper chamber. The lower chamber was filled with complete medium containing 10% FBS and supplemented with TIL-B supernatant. The chambers were incubated at 37 °C in a humidified atmosphere with 5% CO₂ for 48 h. After incubation, non-migrated cells on the upper surface of the membrane were gently removed with a cotton swab. The migrated cells on the lower surface were fixed with 4% paraformaldehyde for 15 min, then stained with 0.1% crystal violet for 20 min. For quantification, five random microscopic fields per insert were counted, and the mean number of migrated cells was calculated.

### Transwell migration assay

The Transwell migration assay was performed using Costar Transwell invasion chambers (Corning, USA). Before the experiment, the Transwell inserts were hydrated with serum-free DMEM medium for 30 min. CD20⁺ B cells (1 × 10⁴) were isolated from the peripheral blood of the same healthy volunteer and resuspended in serum-free medium. Tumor cells derived from non-metastatic or metastatic NPC were seeded into the lower chambers of 24-well plates containing complete medium, and CD20⁺ B cells were subsequently added to the upper chambers of the Transwell inserts. After 24 h of co-culture, the medium was discarded, and the non-migrated cells on the upper membrane surface were gently removed with a cotton swab. The cells that had migrated to the lower surface of the membrane were fixed, stained, and counted under randomly selected high-power fields.

### Animal experiments

Female NSG mice (4 weeks old) were injected via the tail vein with PBMCs from healthy donors (1 × 10⁷/mice) to establish a humanized model. After 7 days, HK1-EBV cells (5 × 10⁶/mice) were subcutaneously inoculated into the right axilla. The mice were randomly divided into three groups: Control group (Hu-PBMC + HK1-EBV), TIL-B group (Hu-PBMC + HK1-EBV, with TIL-B administered via tail vein injection at 1 × 10⁶/mouse, twice weekly), and Anti-CD20 group (Hu-PBMC + HK1-EBV, with intraperitoneal injection of anti-CD20 antibody [clone 2H7, Bio X Cell, BE0276] at 10 mg/kg, twice weekly) (*n* = 4). Tumor volume was measured every 5 days during the experiment. At 28 days, the mice were sacrificed, and tumors were excised, weighed, and photographed. Paraffin-embedded tumor sections were analyzed by IHC for Ki67 and CD20 expression, with positive cells counted in five randomly selected high-power fields (HPF). For the lung metastasis model, mice were injected via the tail vein with HK1-EBV cells (1 × 10⁶/mice), with treatment groups and administration protocols the same as above. After 4 weeks, the mice were sacrificed, visible lung surface nodules were counted, and lung tissues were subjected to Hematoxylin-eosin staining (HE) staining analysis.

### Hematoxylin-eosin staining

Lung tissues fixed in 10% neutral buffered formalin were routinely paraffin-embedded and sectioned at a thickness of 4 μm. Sections were sequentially deparaffinized in xylene, rehydrated through a graded ethanol series, stained with hematoxylin for 5 min, rinsed with tap water, differentiated in 1% acid alcohol for 30 s, blued for 1 min, counterstained with eosin for 2 min, dehydrated through graded ethanol, cleared in xylene, and mounted with neutral resin. The slides were then examined and photographed under a light microscope.

### Immunohistochemistry

Paraffin sections (4 μm) were deparaffinized in xylene and rehydrated through a graded ethanol series, followed by antigen retrieval in citrate buffer (pH 6.0) using a pressure cooker. Endogenous peroxidase activity was blocked by incubation with 3% H₂O₂ at room temperature for 10 min, and nonspecific binding was blocked with 5% BSA at room temperature for 30 min. For IHC staining, primary antibodies [CD20 (Abcam, ab78237), Ki67 (CST, 9449), E-cadherin (CST, 3195), N-cadherin (CST, 13116), and Vimentin (Abcam, ab92547)] were applied and incubated overnight at 4 °C. The next day, HRP-conjugated secondary antibodies were added and incubated at 37 °C for 30 min, followed by DAB chromogenic development and hematoxylin counterstaining. Images were captured using a fluorescence microscope.

### Immunofluorescence

NPC cells were seeded onto sterile coverslips (or culture dishes). Once the cells adhered and reached an appropriate density, they were gently washed twice with PBS, fixed with 4% paraformaldehyde for 15 min, and washed three times with PBS. The cells were then permeabilized with 0.1% Triton X-100 at room temperature for 10 min, washed with PBS, and blocked with 5% BSA at room temperature for 30 min. Primary antibodies [E-cadherin (CST, 3195), N-cadherin (CST, 13116), and Vimentin (Abcam, ab92547)] were applied and incubated overnight at 4 °C. The next day, after equilibrating to room temperature, the cells were washed three times with PBS (5 min each) and incubated with the corresponding fluorophore-conjugated secondary antibodies at 37 °C for 1 h in the dark. After another three PBS washes, the nuclei were counterstained with DAPI (1 μg/mL) for 5 min in the dark, washed with PBS, and mounted using an anti-fade mounting medium. Images were acquired using a laser confocal microscope.

### TUNEL assay

Apoptosis was detected using the In Situ Cell Death Detection Kit (Roche, 11684795910). For tissue sections, samples were treated with proteinase K, followed by incubation with the TdT reaction mixture at 37 °C for 1 h, washed with PBS, counterstained with DAPI, and the proportion of green-positive cells was counted under a fluorescence microscope. For NPC cells, sterile coverslips were seeded with cells and, after attachment, washed twice with PBS, fixed with 4% paraformaldehyde for 30 min, and washed three times with PBS. Cells were then permeabilized on ice for 2 min with 0.1% Triton X-100 containing 0.1% sodium citrate, washed three times with PBS, and incubated with the TUNEL reaction mixture prepared according to the manufacturer’s instructions at 37 °C in the dark for 60 min. After three PBS washes, cells were counterstained with DAPI (1 μg/mL) for 5 min, washed with PBS, and mounted. Images were acquired using a laser confocal microscope with FITC and DAPI channels.

### ELISA assay

The levels of IL-12 (R&D Systems, DY1270), IFN-γ (R&D Systems, DIF50C), TNF-α (R&D Systems, DTA00D), and CXCL13 (R&D Systems, DCX130) were measured using human ELISA kits. Test samples included TIL-B culture supernatants derived from non-metastatic and metastatic NPC, as well as TIL-B culture supernatants isolated from subcutaneous tumors of humanized NSG mice. The assays were performed according to the manufacturers’ instructions, and absorbance was measured at 450 nm. Protein concentrations were calculated based on standard curves.

### Multiplex immunofluorescence imaging

Multiplex immunofluorescence (mIF) staining was performed on 4-μm-thick formalin-fixed, paraffin-embedded (FFPE) NPC tissue sections. The sections were deparaffinized in xylene, rehydrated through graded ethanol, and subjected to heat-induced antigen retrieval in citrate buffer (pH 6.0) at 95 °C for 15 min. Non-specific binding was blocked with 5% normal goat serum at room temperature for 30 min. Primary antibodies against the target proteins from different sources were applied sequentially, each followed by incubation with a specific secondary antibody conjugated to a distinct fluorophore. Fixation and blocking steps were performed between staining cycles to prevent cross-reactivity. Nuclei were counterstained with DAPI, and sections were mounted using an anti-fade mounting medium. All images were acquired using identical parameters on an Akoya multispectral tissue imaging system.

### Vasculogenic mimicry assay

The Matrigel (Corning, 354234) was thawed overnight at 4 °C, and 50 μL was added to a pre-chilled 96-well plate and incubated at 37 °C for 30 min to allow gelation. HK1-EBV or C666-1 cells were digested with trypsin and resuspended in RPMI-1640 medium containing 2% FBS (2 × 10⁴ cells/100 μL). According to the grouping, IL-12 (or control medium) was added before seeding onto the Matrigel-coated wells. The cells were cultured at 37 °C with 5% CO₂ for 8 h, and tube formation was observed and photographed under an inverted microscope.

### CD31/PAS double staining for VM detection

CD31/PAS double staining was performed to identify VM. Paraffin-embedded tumor sections (4 μm) were deparaffinized, rehydrated, and subjected to antigen retrieval. Sections were incubated with anti-CD31 antibody (CST, #77699) followed by DAB detection to visualize endothelial cells. Subsequently, periodic acid-Schiff (PAS) staining (MCE, HY-K0604) was performed according to the manufacturer’s instructions to detect extracellular matrix structures. VM structures were defined as PAS-positive, CD31-negative, channel-like structures containing red blood cells. The number of VM structures was quantified in at least five randomly selected high-power fields per section by two independent observers.

### Statistics

All data are expressed as the mean ± standard deviation (mean ± SD). Comparisons between two groups were performed using a two-tailed Student’s *t*-test, while comparisons among multiple groups were conducted using one-way analysis of variance (one-way ANOVA). Statistical analyses were performed using GraphPad Prism 9.0 software, and *P* < 0.05 was considered statistically significant.

### Ethics approval and consent to participate

This study was approved by the Ethics Committee of the Second Xiangya Hospital of Central South University. Written informed consent was obtained from all patients prior to inclusion. All animal experiments were approved by the Animal Care and Use Committee of Second Xiangya Hospital of Central South University and performed in accordance with institutional and national guidelines.

## Results

### Single-cell transcriptomics and multiplex immunofluorescence reveal abundant TLS structures in tumor regions of non-metastatic NPC

**Fig. 1:**
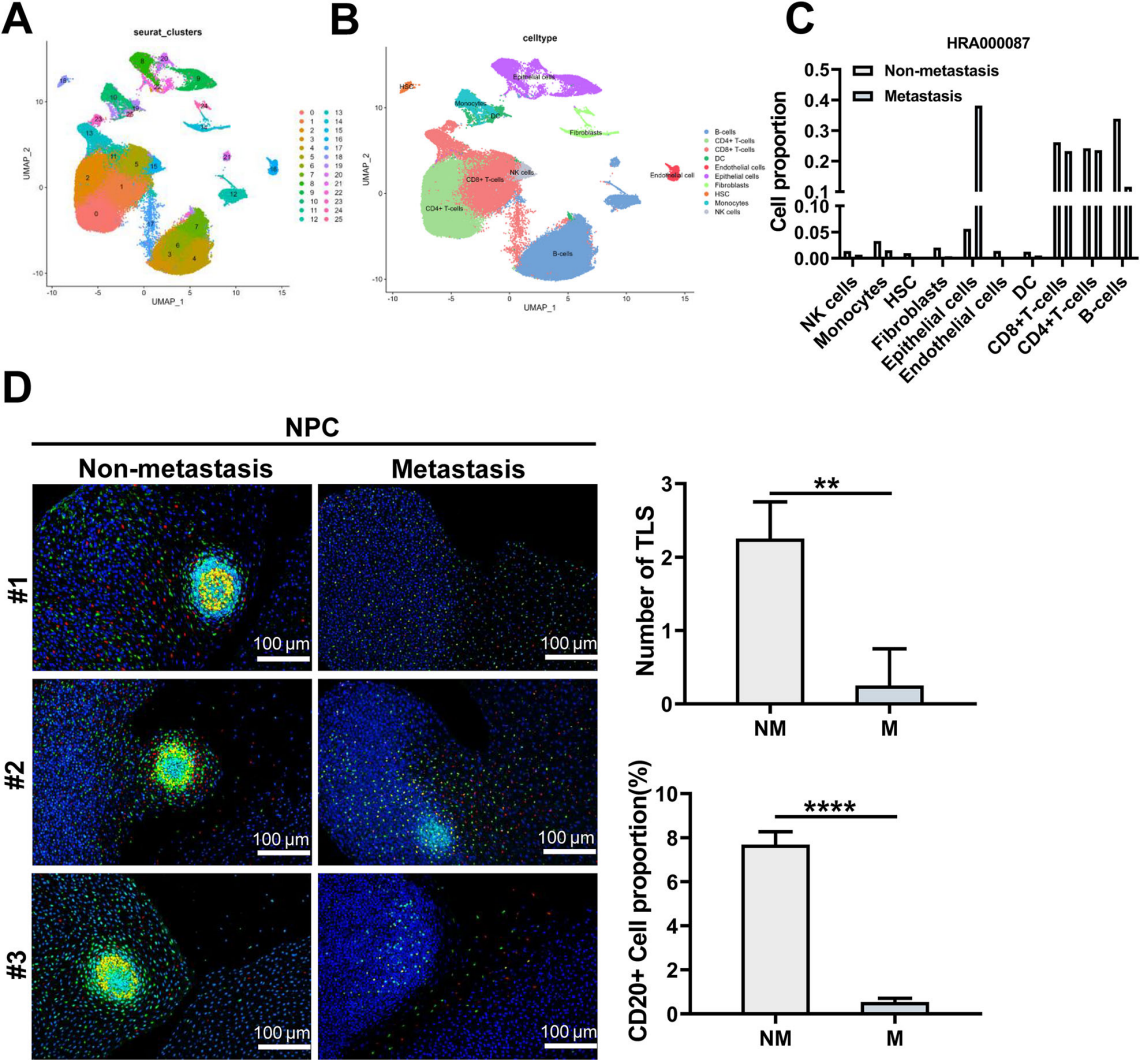
Spatial distribution of B cell aggregation and TLS in non-metastatic nasopharyngeal carcinoma. **A**, **B** Based on single-cell RNA sequencing data from 19 EBV-positive NPC patients (HRA000087), after quality control, dimensionality reduction clustering, and cell type annotation, subpopulations including B cells, CD4⁺ T cells, CD8⁺ T cells, NK cells, dendritic cells (DC), monocytes, hematopoietic stem cells (HSC), fibroblasts, epithelial cells, and endothelial cells were identified. **C** Cell proportion analysis showed that, in addition to T cells, B cells also accounted for a certain proportion. **D** Multiplex immunofluorescence revealed that TLS were only observed in tumor regions of non-metastatic NPC, characterized by clusters of B cells surrounded by T cells, accompanied by distinct B cell follicle structures; whereas in metastatic NPC, B cells were scattered without TLS formation. CD4: green fluorescence, marking helper T cells; CD8: red fluorescence, marking cytotoxic T cells; CD20: yellow fluorescence, marking B cells; CD21: cyan fluorescence, marking B-cell follicles. ns, not significant; *P < 0.05; **P < 0.01; ***P < 0.001; ****P < 0.0001.

To systematically characterize the immune cell composition of the TME in EBV-positive NPC, we analyzed single-cell RNA sequencing data from tumor tissues of 19 NPC patients (dataset HRA000087). Following rigorous quality control, dimensionality reduction, clustering, and cell type annotation, multiple cellular subpopulations were identified, including B cells, CD4⁺/CD8⁺ T cells, NK cells, dendritic cells, monocytes, hematopoietic stem cells, fibroblasts, epithelial cells, and endothelial cells (Fig. 1A, B). Proportional analysis revealed that T cells constituted the predominant immune population, while B cells also accounted for a substantial fraction (Fig. 1C), suggesting their potential functional importance within the NPC immune microenvironment.

To further validate these findings and delineate the spatial distribution of B cells, multiplex immunofluorescence staining was performed on non-metastatic (NM) and metastatic (M) NPC tissues (Fig. 1D). In NM-NPC tumor regions, characteristic tumor-associated TLSs were observed, featuring dense aggregates of CD20⁺ B cells (yellow) surrounded by CD4⁺ (green) and CD8⁺ (red) T cells, along with the presence of CD21⁺ (cyan) follicular structures. Nuclei were counterstained with DAPI (blue). In contrast, typical TLSs were absent in M-NPC tissues, where B cells displayed a dispersed distribution without organized clustering. Further quantitative analysis demonstrated that the number of TLSs was significantly higher in the NM group compared with the M group, accompanied by a markedly increased proportion of CD20⁺ B cells, indicating a strong association between B cell enrichment, TLS formation, and the non-metastatic phenotype.

In summary, these results indicate that TLS formation is significantly negatively correlated with the metastatic status of NPC and is predominantly observed in non-metastatic tumors, suggesting that B cells may mediate local anti-tumor immunity through TLS establishment, thereby playing a critical role in suppressing NPC progression and metastasis.

### Potential role of B cells in inhibiting NPC metastasis and their relationship with tumor cell heterogeneity

**Fig. 2:**
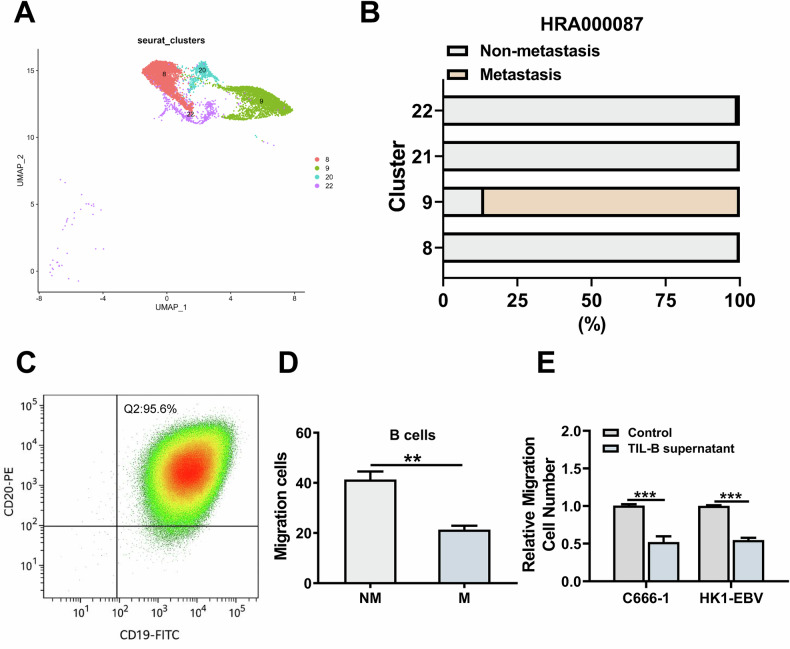
B cells inhibit NPC cell migration and their relationship with tumor cell heterogeneity. **A**, **B** Single-cell transcriptome analysis revealed significant differences in expression patterns between metastatic and non-metastatic NPC tumor cells. **C** MACS-sorted human CD20⁺ B cells were verified by FACS to have a purity >95%. **D** Migration assays showed that NM-NPC cells significantly enhanced the recruitment of peripheral blood B cells, whereas M-NPC cells exhibited weaker recruitment ability. **E** Transwell assays demonstrated that B cell culture supernatant markedly inhibited the migration of C666-1 and HK1-EBV cells. ns, not significant; *P < 0.05; **P < 0.01; ***P < 0.001; ****P < 0.0001.

To investigate the characteristics of B cells in NPC and their regulatory role in tumor metastasis, we first compared the heterogeneity of tumor cells from metastatic and non-metastatic NPC (Fig. 2A, B). Single-cell transcriptomic analysis revealed significant differences in the expression profiles of immune regulation- and migration-related genes between the two groups, suggesting that tumor cells in different metastatic states may exert distinct modulatory effects on immune cells.

Next, CD20⁺ B cells were isolated from the peripheral blood of NPC patients using magnetic-activated cell sorting (MACS). Flow cytometry (FACS) confirmed a purity greater than 95%, meeting the requirements for functional assays (Fig. 2C). Transwell migration assays demonstrated that NM-NPC cells exhibited a markedly higher ability to recruit peripheral B cells compared to M-NPC cells (Fig. 2D), indicating that NM-NPC may enhance local immune responses by promoting B cell infiltration.

Furthermore, we evaluated the effect of B cell-derived soluble factors on NPC cell migration. The results showed that B cell-conditioned medium significantly inhibited the migratory capacity of both C666-1 and HK1-EBV cells (Fig. 2E), suggesting that B cells can suppress the migratory phenotype of NPC through secreted factors.

In summary, the recruitment capacity of B cells in NPC is closely associated with metastatic status, and their secretory products can directly inhibit tumor cell migration, suggesting that B cells may participate in the regulation of NPC metastasis through a “ recruitment-secretion “-related mechanism.

### Both TIL-B cells and their secreted products can inhibit the proliferation of NPC cells

**Fig. 3:**
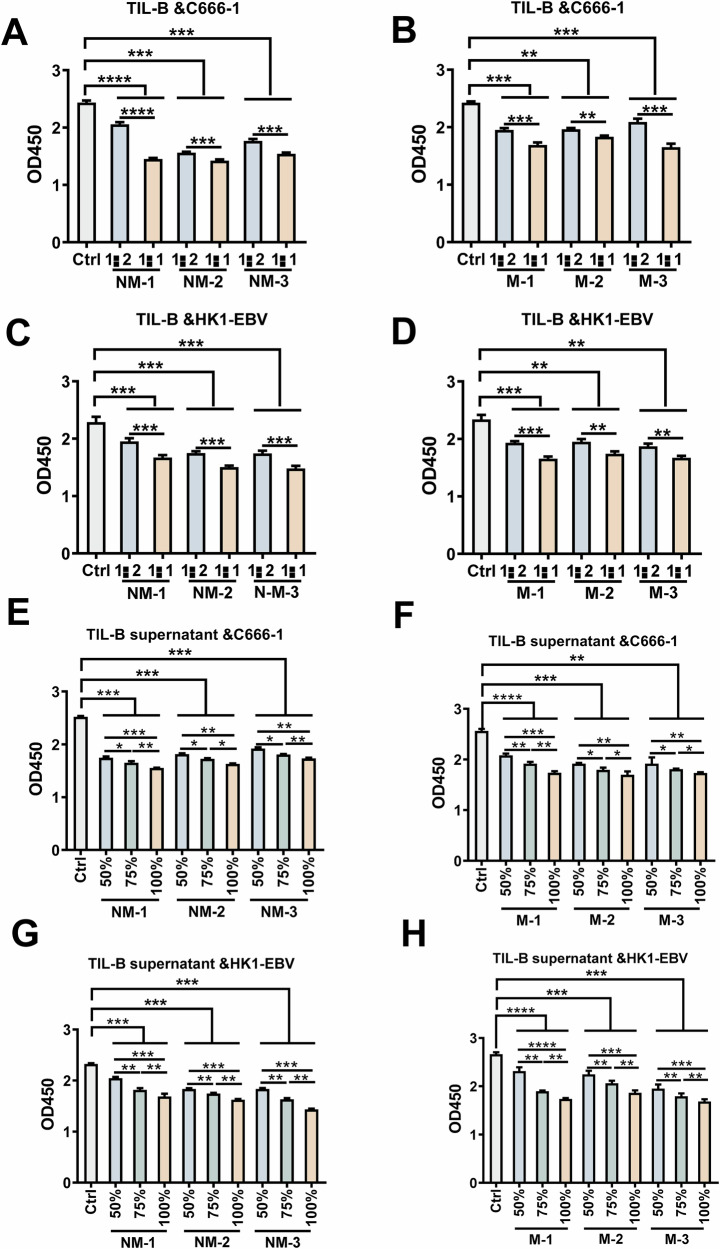
TIL-B cells and their conditioned medium inhibit NPC cell proliferation. **A**–**D** C666-1 and HK1-EBV cells were co-cultured for 48 h with TIL-B derived from non-metastatic or metastatic NPC patients at different N:T ratios (1:2, 1:1). CCK-8 assays showed that TIL-B from both groups significantly reduced the OD₄₅₀ values of NPC cells, with stronger inhibition observed at N: T = 1:1. **E**–**H**. Both NPC cell lines were co-cultured for 48 h with different concentrations (50%, 75%, 100%) of TIL-B conditioned medium. OD₄₅₀ values decreased further with increasing medium concentration, with the highest concentration producing the strongest inhibitory effect. ns, not significant; *P < 0.05; **P < 0.01; ***P < 0.001; ****P < 0.0001.

To evaluate the effect of TIL-B cells on the growth of NPC cells, C666-1 and HK1-EBV cells were co-cultured at different ratios (N: T = 1:2 and 1:1) with TIL-B cells derived from either non-metastatic (NM) or metastatic (M) patients. CCK-8 assays showed that TIL-B cells from both groups significantly reduced the OD₄₅₀ values of NPC cells, with stronger inhibition observed at the N: T = 1:1 ratio (Fig. 3A–D). Furthermore, TIL-B cell-conditioned medium at different concentrations (50%, 75%, and 100%) also inhibited NPC cell proliferation in a concentration-dependent manner, with the highest concentration producing the most pronounced effect (Fig. 3E–H). These results indicate that, regardless of origin from NM or M patients, both TIL-B cells and their secreted products can suppress NPC cell proliferation, suggesting that both direct cell-cell contact and soluble factors may be involved in this process.

### TIL-B cells suppress subcutaneous tumor growth and lung metastasis in humanized nasopharyngeal carcinoma mouse models

**Fig. 4:**
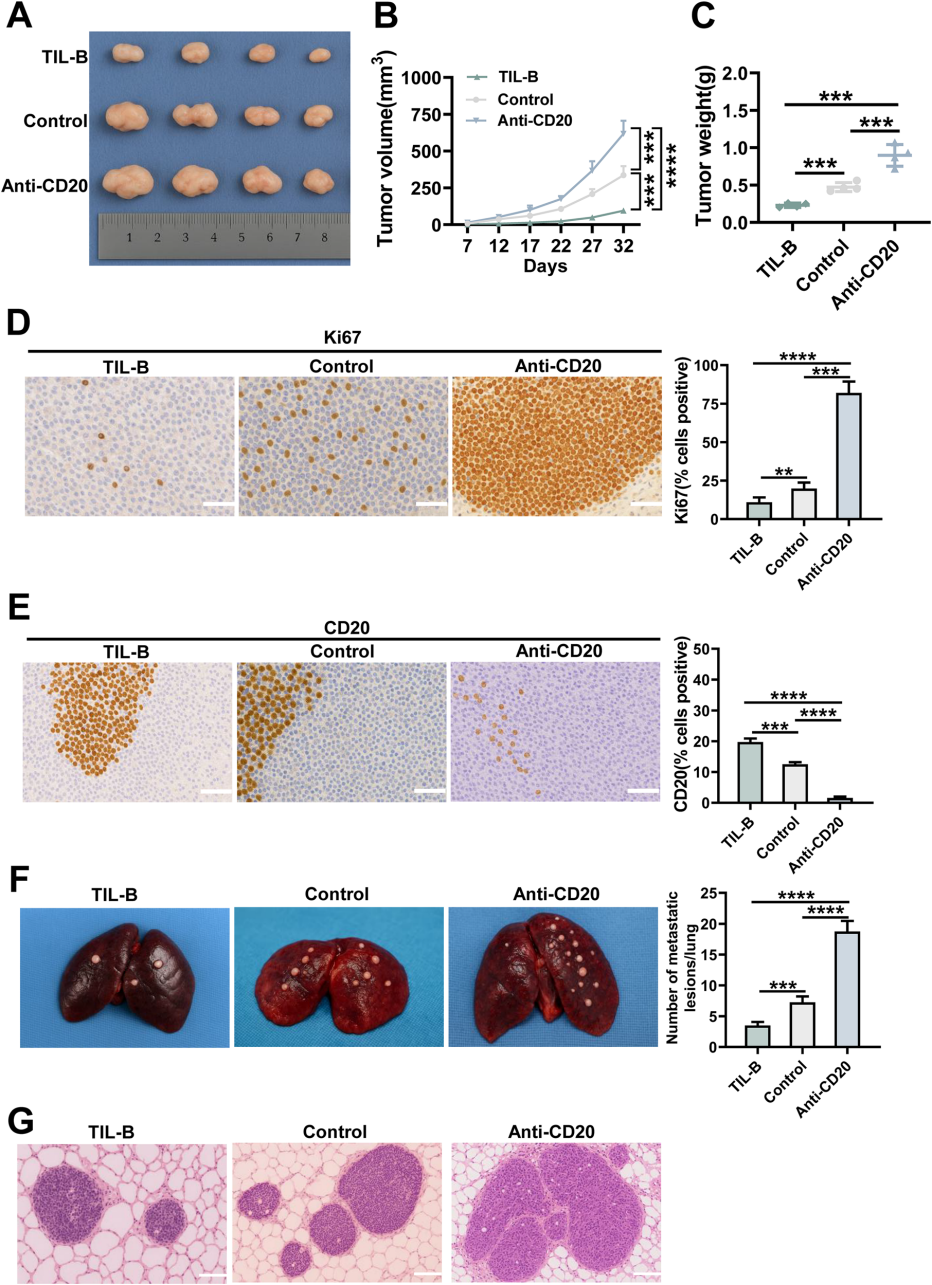
TIL-B cells suppress subcutaneous tumor growth and lung metastasis in humanized NPC mouse models. **A** Schematic of the Hu-PBMC NSG subcutaneous tumor model and representative tumor images. Three treatment groups: Control (Hu-PBMC + HK1-EBV), TIL-B (Hu-PBMC + HK1-EBV + TIL-B cells, intravenous injection), and Anti-CD20 (Hu-PBMC + HK1-EBV + anti-CD20 antibody). **B** Tumor growth curves show significantly slower growth in the TIL-B group and accelerated growth in the Anti-CD20 group. **C** Endpoint tumor weights: lowest in the TIL-B group, highest in the Anti-CD20 group. **D** Ki67 immunohistochemistry of tumor tissues: the TIL-B group exhibited the lowest Ki67-positive rate, while the Anti-CD20 group had the highest. **E** CD20 immunohistochemistry of tumor tissues: the TIL-B group showed the highest B cell infiltration, whereas the Anti-CD20 group showed the lowest. **F** In the lung metastasis model, lung metastatic nodules were markedly reduced in the TIL-B group and increased in the Anti-CD20 group. **G** HE staining revealed that the TIL-B group had small, scarce metastatic foci, whereas the Anti-CD20 group had large metastatic lesions that extensively disrupted alveolar structures. ns, not significant; *P < 0.05; **P < 0.01; ***P < 0.001; ****P < 0.0001.

To evaluate the in vivo effects of TIL-B on NPC growth and metastasis, a humanized Hu-PBMC NSG mouse model was established and divided into three groups: Control, TIL-B (intravenous injection of TIL-B), and Anti-CD20 (intraperitoneal injection of anti-CD20 antibody) (Fig. 4A).

In the subcutaneous xenograft model, both tumor volume and tumor weight were significantly reduced in the TIL-B group compared with the Control group, whereas they were markedly increased in the Anti-CD20 group (Fig. 4B, C), indicating that TIL-B effectively suppress tumor growth, while B cell depletion promotes tumor progression. Consistently, Ki67 immunohistochemistry revealed a pronounced decrease in tumor cell proliferative activity in the TIL-B group, whereas the Anti-CD20 group exhibited enhanced proliferation (Fig. 4D). Moreover, CD20 staining showed the highest level of B cell infiltration in the TIL-B group, followed by the Control group, with the lowest level observed in the Anti-CD20 group (Fig. 4E), consistent with the intervention strategies and corresponding tumor phenotypes. In the lung metastasis model, the number of metastatic nodules was significantly reduced in the TIL-B group but markedly increased in the Anti-CD20 group (Fig. 4F). These findings were further confirmed by HE staining (Fig. 4G), supporting a suppressive role of TIL-B in distant metastasis.

Collectively, these results demonstrate that TIL-B markedly inhibit NPC growth and lung metastasis in vivo, whereas B cell depletion accelerates tumor progression, highlighting a critical tumor-suppressive role of TIL-B in NPC immune defense.

### TIL-B-derived cytokines and their role in regulating apoptosis and proliferation of NPC cells

**Fig. 5:**
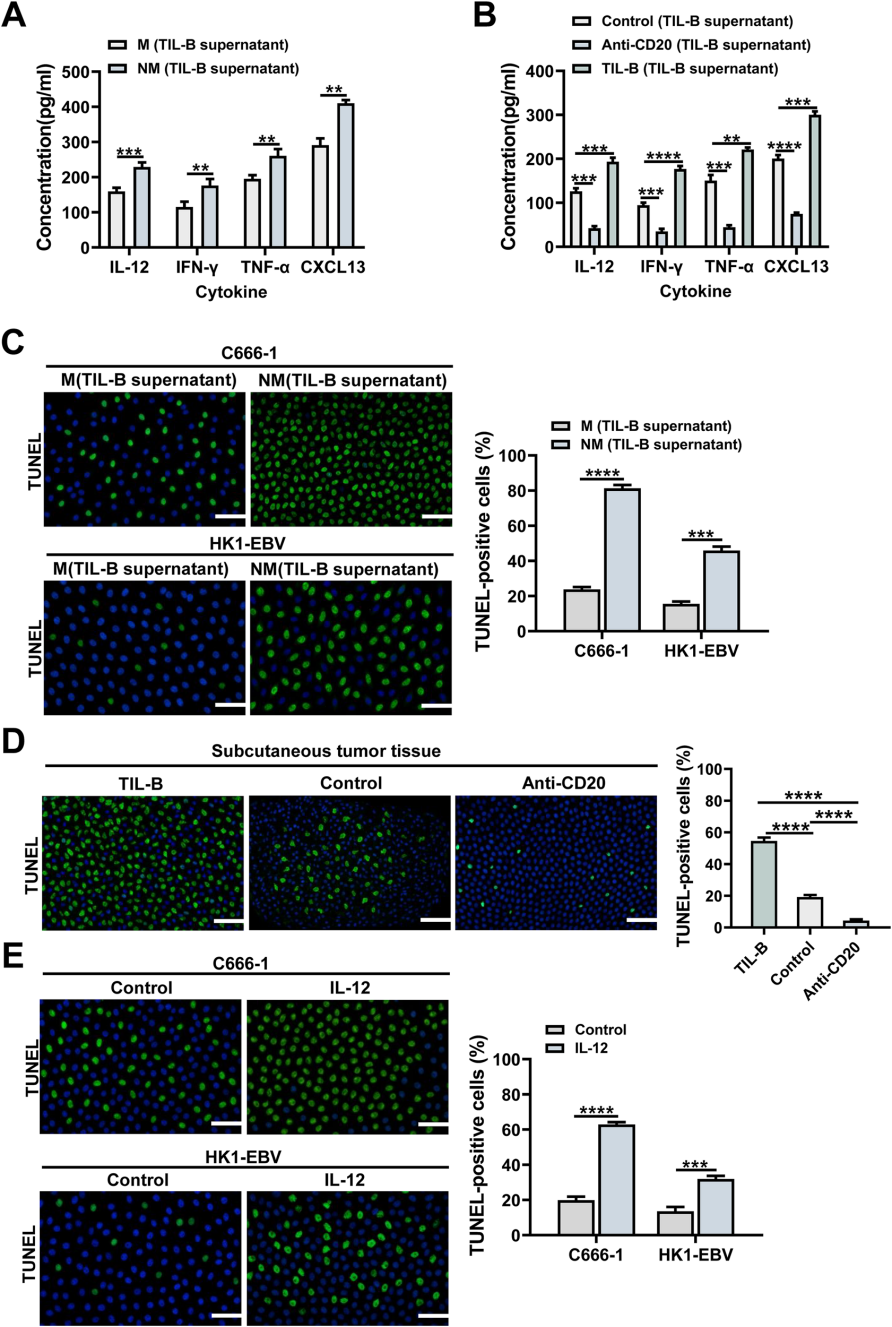
Cytokine secretion and functional analysis of TIL-B cells from NPC patients. **A** ELISA detection of IL-12, IFN-γ, TNF-α, and CXCL13 levels in the culture supernatants of TIL-B cells from NPC patients at different disease stages. **B** Cytokine levels in TIL-B culture supernatants from subcutaneous tumors in humanized NSG mice. **C** Effects of TIL-B supernatants from different sources on NPC cells. **D** TUNEL staining to assess apoptosis in mouse subcutaneous tumor tissues. **E** Recombinant human IL-12 significantly promoted NPC cell apoptosis, with effects comparable to NM-derived TIL-B supernatants. ns, not significant; *P < 0.05; **P < 0.01; ***P < 0.001; ****P < 0.0001.

We first used human ELISA kits to measure the secretion levels of IL-12, IFN-γ, TNF-α, and CXCL13 in the culture supernatants of TIL-B derived from non-metastatic and metastatic NPC patients. The results showed that these cytokines were overall significantly reduced in the M group, with the most pronounced decreases observed in IL-12 and IFN-γ (Fig. 5A), suggesting a potential impairment of the antitumor function of TIL-B in metastatic NPC.

Further analysis in a humanized NSG mouse model revealed that, compared with the TIL-B group, TIL-B derived from the Anti-CD20 group exhibited significantly reduced secretion of IL-12 and CXCL13 (Fig. 5B), indicating that B cell depletion not only reduces cell abundance but also compromises their functional secretory capacity within the tumor microenvironment.

Functional assays further demonstrated that supernatants from NM-derived TIL-B markedly promoted apoptosis and inhibited proliferation of NPC cells, whereas supernatants from the M group, although still exhibiting pro-apoptotic effects, showed substantially weaker activity (Fig. 5C), suggesting limited antitumor capacity likely due to insufficient secretion of key cytokines. Consistently, in vivo TUNEL staining showed that the proportion of apoptotic cells in subcutaneous tumors was significantly higher in the TIL-B group than in the Control and Anti-CD20 groups (Fig. 5D), further supporting that TIL-B induce tumor cell apoptosis through the secretion of pro-inflammatory cytokines.

To further verify the key role of IL-12, recombinant human IL-12 was directly applied to NPC cells. The results demonstrated that IL-12 significantly induced apoptosis and suppressed proliferation, with effects comparable to those of NM-derived TIL-B supernatants (Fig. 5E), thereby functionally confirming IL-12 as a critical effector molecule mediating the antitumor activity of TIL-B.

### TIL-B supernatant and IL-12 inhibit the EMT process of NPC cells and reduce their metastatic potential

**Fig. 6:**
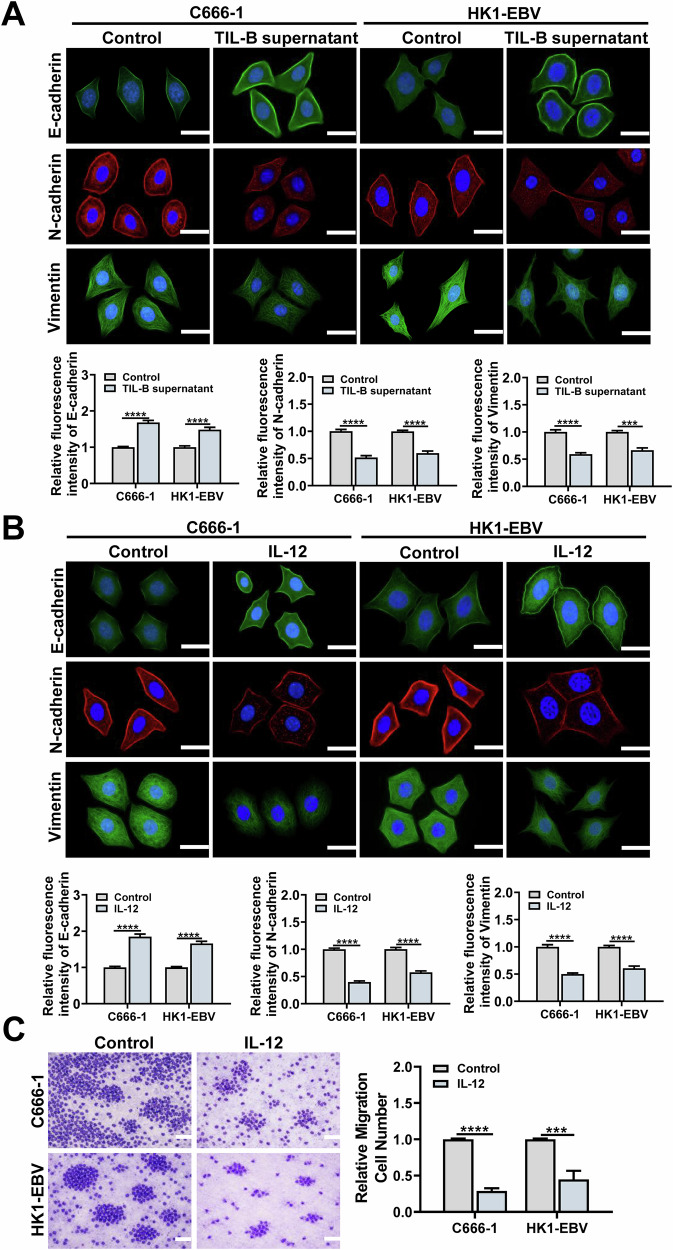
Both TIL-B supernatants and IL-12 suppress EMT and reduce the migratory capacity of NPC cells. **A** C666-1 and HK1-EBV cells were co-cultured with TIL-B culture supernatants for 48 h, and immunofluorescence was used to detect E-cadherin, N-cadherin, and Vimentin protein levels. **B** Treatment with IL-12 for 48 h induced similar changes in EMT marker expression in both NPC cell lines, with increased E-cadherin and decreased N-cadherin and Vimentin. **C** Transwell migration assays showed that IL-12 treatment significantly reduced the number of cells that migrated through the Matrigel-coated membrane. ns, not significant; *P < 0.05; **P < 0.01; ***P < 0.001; ****P < 0.0001.

To evaluate the effects of TIL-B-derived secretory products on EMT and migratory capacity of NPC cells, C666-1 and HK1-EBV cells were co-cultured with TIL-B-conditioned medium, and EMT-related markers were assessed (Fig. 6A). The results showed that treatment with TIL-B supernatants significantly upregulated the epithelial marker E-cadherin while markedly downregulating the mesenchymal markers N-cadherin and Vimentin, indicating suppression of the EMT process.

To further identify the key effector molecules involved, we performed functional validation of IL-12. Exogenous IL-12 treatment partially recapitulated the effects of TIL-B-conditioned medium, as evidenced by increased E-cadherin expression and decreased N-cadherin and Vimentin levels, along with a significant reduction in cell migratory capacity (Fig. 6B, C), suggesting that IL-12 contributes to the anti-EMT effects mediated by TIL-B.

To further determine the functional contribution of IL-12, neutralizing antibodies against IL-12 were applied to TIL-B-conditioned medium. Blocking IL-12 partially reversed the EMT-suppressive effects induced by TIL-B supernatants, as reflected by a reduction in E-cadherin upregulation and a partial restoration of the decreased N-cadherin and Vimentin levels (Fig. S1A). Consistently, cell migratory capacity was partially restored (Fig. S1B), although these effects did not fully return to control levels.

Collectively, these findings indicate that TIL-B-derived secretory factors markedly suppress EMT and reduce the migratory capacity of NPC cells. IL-12 plays an important, but not exclusive, role in this process, suggesting that TIL-B regulate EMT through a coordinated, multi-factorial mechanism, thereby limiting the invasive and metastatic potential of NPC cells.

### TIL-B suppresses EMT in vivo, and IL-12 inhibits vasculogenic mimicry in NPC

**Fig. 7:**
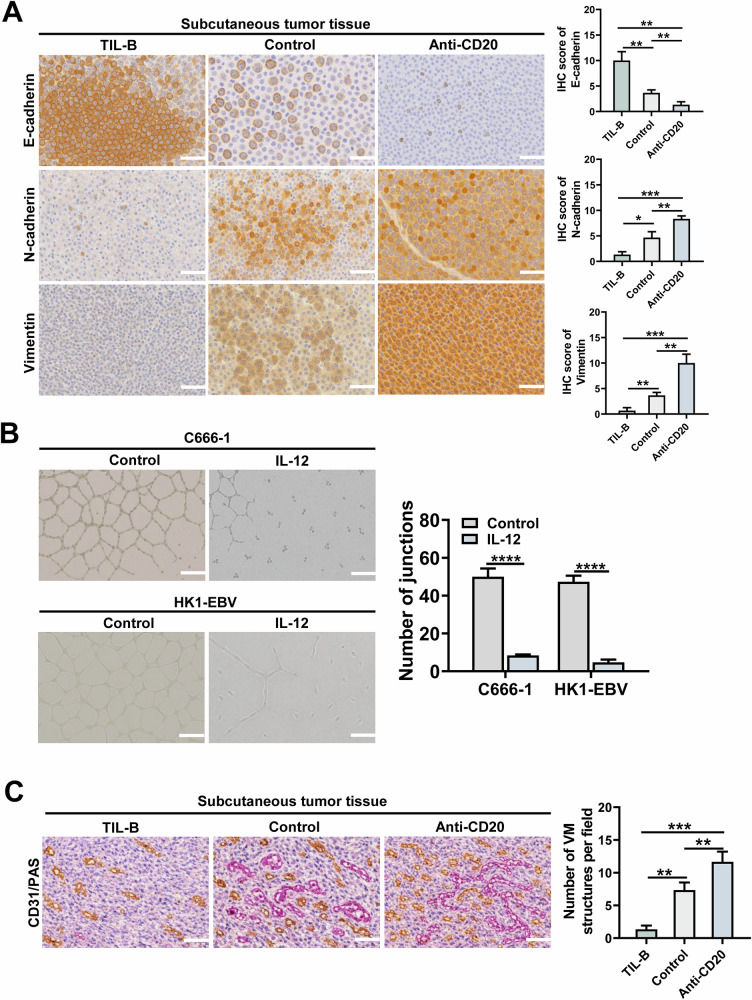
EMT and VM analyses in NPC models. **A** IHC staining of E-cadherin, N-cadherin, and Vimentin in tumors from the Hu-PBMC NSG subcutaneous xenograft model (Control, TIL-B, and Anti-CD20 groups). Nuclei were counterstained with hematoxylin (blue-purple). **B** Matrigel-based three-dimensional tube formation assays of C666-1 and HK1-EBV cells with IL-12 treatment. **C** CD31/PAS double staining of subcutaneous tumor tissues. CD31 (brown) labels endothelial cells, and PAS (magenta) highlights VM-associated structures. VM was defined as PAS-positive and CD31-negative channel-like structures. Quantification of VM structures per field is shown. ns, not significant; *P < 0.05; **P < 0.01; ***P < 0.001; ****P < 0.0001.

To further delineate the in vivo impact of TIL-B on EMT in NPC, immunohistochemical analyses of EMT markers were performed in tumor tissues from a Hu-PBMC NSG subcutaneous xenograft model (Fig. 7A). Relative to the Control group, TIL-B treatment was associated with a pronounced upregulation of E-cadherin and a concomitant reduction in N-cadherin and Vimentin expression, indicative of EMT attenuation in vivo. In contrast, B cell depletion via anti-CD20 resulted in decreased E-cadherin expression together with increased N-cadherin and Vimentin levels, demonstrating an opposing pattern and supporting a role for B-cell loss in promoting EMT progression.

We next evaluated the effect of IL-12 on VM in NPC cells using in vitro assays. In Matrigel-based three-dimensional tube formation assays, IL-12 treatment led to a significant reduction in the formation of canonical VM-like networks in both C666-1 and HK1-EBV cells, as evidenced by decreased network density, impaired structural integrity, and reduced branching (Fig. 7B), indicating suppression of VM-forming capacity.

To assess VM in vivo, CD31(brown)/PAS (magenta) dual staining was performed on xenograft tumor sections. In the Control group, typical VM structures were identified as PAS-positive (magenta), CD31-negative patterned channels. These structures were markedly reduced in the TIL-B group. In contrast, the Anti-CD20 group exhibited a substantial increase in VM structures, characterized by abundant PAS-positive, CD31-negative channels (Fig. 7C).

Taken together, these data indicate that TIL-B is associated with suppression of EMT and reduction of VM structures in vivo, while in vitro evidence supports an inhibitory effect of IL-12 on VM formation. Given the established roles of EMT and VM in tumor invasion and metastatic dissemination, these findings suggest that TIL-B may constrain NPC metastatic potential through coordinated regulation of invasion-associated phenotypes, potentially mediated by its secreted factors, including IL-12.

## Discussion

This study is the first to systematically elucidate the distribution patterns, functional roles, and molecular mechanisms of TIL-Bs in NPC. By integrating single-cell RNA sequencing, histological analyses, and both in vitro and in vivo functional assays, we demonstrate that TIL-Bs are not passive bystanders within the tumor immune microenvironment but rather key effector cells that actively regulate tumor progression through multidimensional mechanisms. At the spatial level, B cells were markedly enriched, and TLSs were well organized in non-metastatic NPC, whereas TLSs were absent and B cells exhibited a dispersed distribution in metastatic NPC. This distinction suggests a close association between TIL-Bs, their structural organization, and tumor metastatic potential. Previous studies have shown that TLSs serve as critical platforms for local antigen presentation and lymphocyte activation and are associated with favorable prognosis in multiple solid tumors [13]. In the present study, CXCL13 was significantly elevated in the non-metastatic group; together with its role in recruiting CXCR5⁺ B cells and follicular helper T (Tfh) cells to promote germinal center–like reactions [14, 15], these findings suggest that the CXCL13–TLS axis may represent a key mechanism for maintaining local immune surveillance and restraining tumor progression [5].

At the functional level, TIL-Bs exhibit pronounced direct anti-tumor effects. Regardless of whether they were derived from non-metastatic or metastatic patients, TIL-Bs significantly inhibited NPC cell proliferation and migration and induced apoptosis. Notably, their conditioned medium produced similar effects, indicating that soluble secreted factors play a central role in this process. Further analyses revealed markedly elevated levels of IL-12, IFN-γ, TNF-α, and CXCL13 in TIL-B supernatants, among which IL-12 and IFN-γ showed the strongest association with the observed anti-tumor effects.IL-12 induces Th1-type immune responses via the STAT4 signaling pathway, promoting CD8⁺ T cells and NK cells to secrete IFN-γ, thereby enhancing anti-tumor cytotoxicity [16]. Meanwhile, IFN-γ stabilizes the epithelial phenotype and restrains tumor progression by suppressing EMT-related transcription factors such as Snail and Twist and downregulating pro-angiogenic molecules [17, 18]. Collectively, these findings indicate that TIL-Bs not only exert indirect anti-tumor effects through immune modulation but also directly influence tumor cell behavior via a cytokine-mediated regulatory network.

Further mechanistic analyses revealed that TIL-Bs exert anti-metastatic effects by coordinately regulating two key processes: EMT and VM. EMT endows tumor cells with enhanced plasticity and invasiveness, whereas VM provides an alternative, endothelial-independent blood supply route, constituting a critical structural basis for tumor metastasis [19, 20]. In this study, TIL-Bs were found to upregulate E-cadherin while downregulating N-cadherin and Vimentin, thereby suppressing the EMT program. Concurrently, IL-12 markedly reduced both the number and integrity of VM structures, suggesting that it not only limits tumor cell migratory capacity but also impairs tumor survival and dissemination by disrupting aberrant vascular-like networks. This coordinated “EMT inhibition +VM blockade” mechanism intervenes in metastasis at both the level of tumor cell phenotype and microenvironmental support, underscoring its important biological significance.

Animal experiments further validated the anti-tumor effects of TIL-Bs in vivo. In a humanized mouse model, TIL-Bs markedly suppressed tumor growth and reduced lung metastasis, whereas B-cell depletion (Anti-CD20 group) significantly accelerated tumor progression. These findings not only provide direct in vivo evidence of the anti-tumor activity of TIL-Bs but also highlight the indispensable role of B cells in maintaining immune homeostasis within the tumor microenvironment. From an immune network perspective, TIL-Bs may exert their effects through a coordinated regulatory mode integrating “cytokine-driven signaling and structural support. “On the one hand, they secrete key cytokines such as IL-12 and IFN-γ to enhance the effector functions of T cells and NK cells; on the other hand, they promote TLS formation via CXCL13, thereby providing a spatial platform for efficient cooperation among multiple immune cell populations and amplifying the overall anti-tumor immune response.

Based on the above multi-level evidence, we propose a “dual-pathway model” by which TIL-Bs mediate anti-metastatic effects in NPC. Specifically, the IL-12–IFN-γ axis acts directly on tumor cells to coordinately suppress EMT and VM, thereby reducing tumor invasiveness and metastatic potential. Meanwhile, the CXCL13–TLS axis remodels the tumor immune microenvironment, enhancing local lymphocyte activation and immune surveillance. These two pathways are synergistically coupled at the levels of intrinsic tumor cell regulation and immune microenvironment remodeling, enabling TIL-Bs to exert both direct effector functions and indirect regulatory roles. Compared with T cells, which primarily rely on cytotoxic activity, TIL-Bs exhibit a composite role as both “structural organizers” and “functional amplifiers”: they not only regulate tumor cell plasticity but also orchestrate local immune ecology, thereby acting as a critical hub in suppressing NPC metastasis.

Although this study reveals the anti-tumor roles of TIL-Bs at multiple levels, several limitations should be acknowledged. First, the relatively limited clinical sample size may affect the robustness and generalizability of the conclusions. Second, the functional heterogeneity of B-cell subsets has not been systematically characterized, and the specific roles and dominant effects of different phenotypic B-cell populations in NPC progression remain to be clarified. In addition, the upstream regulatory network of IL-12 and CXCL13, as well as their dynamic interactions with other immune components, is not yet fully understood. Therefore, future studies integrating single-cell multi-omics, spatial transcriptomics, and functional perturbation experiments are warranted to achieve a more refined and causal dissection of the TIL-B–mediated immune regulatory network, thereby further strengthening the mechanistic foundation of this study.

From a translational perspective, our findings provide important implications for immunotherapeutic strategies in NPC. The protective role of TIL-Bs suggests that indiscriminate B-cell depletion may carry potential risks in this context, and its clinical application should be carefully stratified based on the specific immune landscape. In contrast, maintaining or selectively enhancing beneficial B-cell functions may represent a more effective therapeutic approach. Importantly, IL-12 and CXCL13 identified in this study not only act as key effector molecules but also offer actionable targets for intervention. Enhancing IL-12–related signaling or promoting CXCL13-mediated TLS formation may amplify local anti-tumor immune responses and strengthen immune synergy within the tumor microenvironment. Overall, these findings support the development of strategies centered on B-cell functional reprogramming or TLS promotion, providing a new theoretical basis and potential avenue for context-dependent precision immunotherapy in NPC.

## Supplementary information

Table S1

Supplemental Figure 1

## Data Availability

All data obtained in this study are available from the corresponding author upon request.
