# Supplementary material for: Tumor-infiltrating B cells inhibit nasopharyngeal carcinoma metastasis
Source: Cell Death Dis. 2026 Jun 2;17(1):689. doi: 10.1038/s41419-026-08940-6 (PMC13444244; doi:10.1038/s41419-026-08940-6)
Supplement: Supplementary file 1 [file 41419_2026_8940_MOESM2_ESM.docx]

**Tumor-infiltrating B cells inhibit nasopharyngeal carcinoma metastasis**

**Fig. S1** **TIL-B suppresses EMT and migration of NPC cells in an IL-12-dependent manner. A.** Immunofluorescence staining of EMT markers: E-cadherin, N-cadherin and Vimentin. TIL-B supernatant increased E-cadherin and decreased N-cadherin and Vimentin, which were partially reversed by IL-12 blockade. **B.** Representative Transwell images and quantification in C666-1 and HK1-EBV cells. TIL-B supernatant significantly inhibited cell migration, which was partially restored by IL-12 neutralization. ns, not significant; **P* < 0.05; ***P* < 0.01; ****P* < 0.001; *****P* < 0.0001.
